# Supplementary material for: SARS-CoV-2 genotyping and sequencing following a simple and economical RNA extraction and storage protocol
Source: PLoS One. 2023 Jan 19;18(1):e0280577. doi: 10.1371/journal.pone.0280577 (PMC9851494; doi:10.1371/journal.pone.0280577)
Supplement: S1 File — (PDF) [file pone.0280577.s001.pdf]

SARS-CoV-2 genotyping and sequencing following a simple and economical RNA Extraction and Storage protocol

Supplemental Material

Sarah Hernandez, Phuong-Vi Nguyen, Taz Azmain, Anne Piantadosi, Jesse J. Waggoner

Table S1. Ct values following RNA extraction with the SARS-RNAES protocol and a commercial extraction robot.

Sample Code	Packet Replicate 1	Packet Replicate 2	Average Packet Ct	Commercial Extraction Ct
1	36.67	33.69	35.18	29.73
2	30.09	28.78	29.44	25.85
3	29.54	31.55	30.55	28.53
4	30.00	29.35	29.68	26.84
5	N	N	--	31.28
6	36.73	N	36.73*	30.64
7	24.45	28.12	26.29	23.23
8	25.75	25.47	25.61	22.38
9	26.14	26.51	26.33	21.83
10	27.45	25.01	26.23	22.72
11	31.35	32.75	32.05	29.45
12	27.54	25.84	26.69	23.51
13	27.24	27.94	27.59	26.09
14	26.44	27.42	26.93	21.64
15	26.86	26.48	26.67	24.18
16	23.66	24.14	23.90	20.36
17	N	25.48	25.48*	17.83
18	39.80	37.33	38.57	29.49
19	22.92	23.40	23.16	20.82
20	23.75	23.31	23.53	20.66
21	31.58	30.84	31.21	24.77
22	32.25	31.60	31.93	27.20
23	25.50	25.11	25.31	21.97
24	24.14	22.92	23.53	21.73
25	30.56	28.76	29.66	26.34
26	30.81	28.65	29.73	20.53

27	25.60	25.19	25.40	22.15
28	27.35	27.82	27.59	24.89
29	35.33	31.85	33.59	29.13
30	35.89	31.45	33.67	28.93

*Average value reflected from one RNAES replicate

N indicates negative

“--” indicates no data available

Table S2. Average concentration of SARS-CoV-2 RNA (log_{10} copies/μL) in duplicate RNAES extractions following ambient temperature storage for 0-, 1-, 3-, and 7-days post extraction.

Sample Code	Day 0	Day 1	Day 3	Day 7
3426	0.32	0.50	0.29	0.09
3531	2.58	2.27	2.40	2.41
3985	1.36	1.46	1.45	0.51
6760	2.61	2.02	2.53	2.03
9268	1.61	0.89*	0.97	0.53

"*" Concentration calculated from a single data point. One of two replicates failed.
