# Supplementary material for: SARS-CoV-2 genotyping and sequencing following a simple and economical RNA extraction and storage protocol
Source: PLoS One. 2023 Jan 19;18(1):e0280577. doi: 10.1371/journal.pone.0280577 (PMC9851494; doi:10.1371/journal.pone.0280577)
Supplement: Attachment — Submitted filename: Response to Reviewers.docx [file pone.0280577.s002.docx]

**Reviewer 1**

**Summary:**

**The authors have done an interesting work and have tried to come up with an alternative and economical solution for RNA extraction and short-term storage at ambient temperature for SARS-CoV-2. Additionally, they have also tried to evaluate the quality of the RNA isolated by their method for SNP detection and WGS.**

We thank Reviewer 1 for the thoughtful comments.

**The paper looks well written and structured, but I feel that it needs to shift the focus of introduction and discussion from the RNA extraction to SARS-CoV-2 genotyping and sequencing. The article in its current form gives a feeling of redundancy and replication of their recently published article “Simple and Economical Extraction of Viral RNA and Storage at Ambient Temperature” and little importance to the novel part of the work.**

Thank you for these comments. We have now significantly edited the Introduction and reorganized the Discussion to emphasize new elements of this article, including the use of eluates for SARS-CoV-2 genotyping in the Spike-SNP assay and whole genome sequencing.

The significantly revised portion of the Introduction (lines 54-61) now reads,

“Using residual clinical samples collected from Autumn 2020 through Spring 2022, this protocol was evaluated for extraction of SARS-CoV-2, and the quality of eluted RNA was further evaluated using genotyping rRT-PCRs that detect single nucleotide polymorphisms (SNPs) in the spike gene and a common whole genome sequencing protocol. Genotyping and sequencing are essential for monitoring the evolution of pathogenic viruses yet require larger amplicons than standard detection assays, and such methods were not evaluated during the initial development of the RNAES protocol.^”^

**Also, it would be better if the extraction protocol is explained properly in the method section of the article because RNA extraction method is one of the aims of work. Other than the writing part, I have few other concerns and questions related to the experiments.**

We added necessary details to work from the methods in this report without having to refer to the original paper. The revised portion (lines 67-76) now reads,

“RNAES extraction packets were assembled with a 5.56-mm diameter membrane disk sandwiched between a square blotter pad base (25 x 25 x 2.5 mm; VWR International, Radnor, PA) and a Parafilm cover (Research Products International, Mt. Prospect, IL) with a 3.96-mm diameter aperture centered over the membrane. Briefly, the protocol consisted of incubating 25μL of respiratory swab sample in 25μL of lysis mixture (150 mM sucrose, Boston BioProducts, Ashland, MA; 5μg proteinase K, New England Biolabs, Ipswich, MA; 2.5μg carrier RNA, Qiagen, Germantown, MD; 100 mM KCl; and 50mM Tris-HCl, pH 7.0, MilliporeSigma) for 10 minutes. Following incubation, 100μL of arginine binding buffer (100 mM L-Arginine; 400 mM KCl, both from MilliporeSigma)/150μL ethanol mixture was combined with the lysate and run dropwise through 5.56mm circular filter membranes.”

**I am surprised that why GF/D membranes, which had best yield for DENV-positive serum samples, didn’t work at all for the SARS-CoV-2 nasopharyngeal samples. The authors have tried to find the reason, but I have few general questions related to it. Was there no RNA in the elution or the PCR didn’t work? How many times did they repeat the experiment with all the three membranes? Did they use the same set of samples and conducted the experiments at the same time?**

Thank you for these questions. We have evaluated RNA extraction with a variety of membranes during the development of this protocol. GF/D, Whatman 3, and Fusion 5 membranes were repeatedly evaluated for SARS-CoV-2 RNA binding and release, and these were always tested at the same time and using the same starting material. We evaluated the protocol with segments of the SARS-CoV-2 genome, purified genomic RNA, and residual clinical samples. RNA was recovered only from packets assembled with Fusion 5 membranes. No RNA was detectable in eluates from other membranes. This RNA may have been bound to the membrane and simply not eluted, or it may not have bound these membranes well. As the reviewer noted, we studied potential causes for this finding but were unable to resolve it with these experiments.

**One of the major claims of their work is the stability of the RNA at ambient temperature for 7 days. It would be worth knowing the difference in the Ct values for the samples eluted at different time points and their comparison with the MagMaxViral RNA Isolation Kit on day 0, for its practical applicability. Have they tested the maximum time limit for successful RNA extraction? The authors have shown that Ct values < 30 yielded genomes with 99-100% coverage. Was there any correlation between the RNA concentration and the Ct values of the samples?**

Thank you for these questions. For comparisons of RNA stability, we calculated the concentration of RNA in the eluate as this shows less run-to-run variability relative to Ct values. That is shown in Figure 2 at box-and-whisker plots, and the values are provided in Supplementary Table 2. We do not typically perform these calculations for SARS-CoV-2, and such calculations were not performed for the larger clinical evaluation of the protocol as those samples were extracted and tested side-by-side on a single rRT-PCR run. However, in response to the last question, the SARS-CoV-2 rRT-PCR is quantitative, and these is a strong, indirect correlation between Ct value and RNA concentration in the sample (higher Ct values result from lower RNA concentrations). Any difference in Ct values between the RNAES protocol and the MagMax Viral protocol would be reflected in the calculated viral load. We have not yet determined the maximum duration of time for successful RNA extraction after ambient temperature storage. Those experiments are ongoing.

**Reviewer 2**

**First, I would like to congratulate the authors for the manuscript, it is well written and deals with a scientific relevant subject. This protocol may help laboratories around the world with limited resources to rapidly detect SARS-CoV-2 in nasopharyngeal samples, specially when facing emergencial situations.**

We appreciate these comments from Reviewer 2.

**The main objective of the research was clear to me and the performed tests showed satisfying results. The comparison with commercial kits revealed the protocol to be consistent and the limitations of the work were properly highlighted. The only issue I had was with Table 1 "Genotypes detected in the Spike SNP assay following RNAES extraction.", it seems it is not well formatted. I kindly ask for the authors to take a look at it.**

We appreciate the thoughtful feedback from Reviewer 2. We have reformatted Table 1 to improve the interpretability of these results.
